# Could contact lens dryness discomfort symptoms sometimes have a neuropathic basis?

**DOI:** 10.1186/s40662-021-00236-4

**Published:** 2021-04-06

**Authors:** Charles W. McMonnies

**Affiliations:** School of Optometry and Vision Science, Faculty of Medicine and Health, University of New South Wales, 77Cliff Avenue, Northbridge Sydney, 2063 Australia

**Keywords:** Contact lens, Symptoms, Neuropathy, Sub-basal nerve plexus

## Abstract

Symptoms of dryness discomfort in soft contact lens wearers frequently lead to discontinuation from wear. The negative influence of pre-fitting tear dysfunctions appears likely to be exacerbated by the challenges to tear homeostasis caused by contact lenses. The corneal mechanisms for symptoms in contact lens wearers are different to those for dry eye disease because the cornea is insulated by the lens from ambient conditions as well as from lid wiper friction during blinking. Symptoms of dryness discomfort might be the consequence of increased lid wiper friction during blinking when the lens front surface becomes soiled and dry and exhibits very rapid tear break up. It is possible that some cases of contact lens intolerance and discontinuation could be a function of lid wiper neuropathy. In relation to the possibility of corneal neuropathy, a stagnant post-lens tear pool with the possibility of increased concentrations of metabolic by-products, cellular debris, and bacterial exotoxins, might have the potential to disturb the corneal epithelial and sub-basal nerves. Contributions by contact lens-induced inflammation to any neuropathic changes may partly depend on the degree to which inflammatory mediators are concentrated in a stagnant post-lens tear pool. It does not appear to be known if corneal neuropathic changes could develop under these conditions. The chances of neuropathic involvement may be greater if discomfort develops after a significant period of successful wear and there is a history of comorbid pain conditions. Esthesiometry and in vivo confocal microscopy in discontinued contact lens wearers may support a diagnosis of contact lens-related corneal neuralgia.

## Background

Findings of a poor correlation between symptoms and signs of dry eye disease and/or lack of satisfactory responses to treatment for tear dysfunction [1], warrant consideration of alternative pathophysiological mechanisms that better explain these apparent paradoxes [2]. The corneal nerves are responsible for sensations of touch, pain, and temperature and they play an important role in the blink reflex, wound healing as well as in tear production and secretion [3] so that dry-eye-like symptoms without signs of tear dysfunction could be due to neuropathic changes in corneal nerves [4–6]. Disruption of corneal nerves with associated interruption of neural feedback loops between the ocular surface and lacrimal glands can lead to dry eye disease [7]. Multiple lines of evidence suggest that peripheral and central neuronal sensitisation processes are involved in generating and maintaining symptoms of dryness in ocular surface disease [8]. That underlying causes of neuropathic corneal pain include contact lenses [9] and chronic symptoms of dryness discomfort, especially when there is a lack of correlation between signs and symptom of dryness [10], prompted this review which examines how peripheral and/or central neuropathic mechanisms could sometimes help explain discomfort symptoms and loss of ability to continue wearing soft contact lenses.

## Main text

### Corneal neuropathic pain

Corneal neuropathic pain, which is also termed corneal neuralgia and corneal allodynia, remains an ill-defined entity [9]. The diagnosis of neuropathic pain requires confirmation of injury or disease affecting somatosensory pathways of the peripheral and/or central nervous systems [9]. Although neuropathic ocular pain evaluation and treatment should be considered for some dry eye patients, this aspect of dry eye management is not routinely examined or treated in the clinical setting [11]. The same limitation applies to the management of contact lens intolerance.

### Soft contact lens symptoms

Contact lens care system-related discomfort symptoms can usually be avoided by changing to a different system of care [12] or to daily disposable lenses. Excess movement of a loose lens fitting may contribute to discomfort and such problems can usually be eliminated by changing lens specifications [10] when a tighter lens fitting with closer conformity to the bulbar conjunctiva has been found to improve comfort [13]. Nevertheless, as discussed below, tighter fittings may be more likely to have implications for symptoms due to reduced tear exchange with associated greater stagnation of post-lens tears. Discomfort and dryness can be prime reasons for contact lens wear discontinuation [14]. Other common reasons for discontinuation include poor vision [10] and contact lens-induced papillary conjunctivitis which is associated with symptoms of excessive lens movement and awareness, itch and excess mucus production [15]. The prevalence of poor vision may be increasing with growing interest in the contact lens correction of astigmatism and presbyopia [10] while the prevalence of contact lens-induced papillary conjunctivitis may be decreasing with the greater use of daily disposable lenses [16].

Dryness is the most common symptom of discomfort in contact lens wearers, with related symptoms of grittiness, scratchiness and foreign body sensations also being very frequently reported [17]. These symptoms seem to be predominantly a problem of poor front lens surface lubricity and wetness [10, 13] when, compared to non-contact lens wearing eyes, increased friction between lid wiper and a poorly lubricated lens surface may explain them. Increased lid wiper friction appears likely to increase the risk of tissue damage. Compared to a desquamating ocular surface in non-contact lens wearers, a non-desquamating contact lens surface appears likely to generate much greater lid wiper friction. For instance, 80% of ‘dryness symptomatic’ soft contact lens wearers showed lid-wiper epitheliopathy stain while only 13% without dryness symptoms showed staining [18]. In addition, the lid wiper epitheliopathy in symptomatic contact lens wearers was graded to be significantly more intense than that found in non-lens wearers [18]. Given that front surface evaporation continues throughout interblink intervals, the key to lid wiper friction and symptom generation appears to be the severity of lens front surface tear film anomaly at the time of a blink when evaporation has peaked [19]. Even a thin tear layer can provide hydrodynamic lubrication but unwetted break up areas lead to boundary lubrication and increased lid wiper friction [19]. Apart from increased evaporation rates due to lipid deficiencies, unwetted break up areas may be due to low blink rates and/or high incomplete blink rates and associated over exposure during longer interblink intervals, especially for the inferior ocular or lens surface. The high proportion of lid wiper stain in symptomatic contact lens wearers might be explained by the degree to which surface deposits accumulate, tear break times shorten and larger areas of unwetting develop on lens front surfaces so that associated reduced surface lubricity provides a higher frictional load for the lid wiper during a blink [19].

### Role of tear dysfunctions

All soft contact lens materials examined have been found to adversely affect tear physiology [20] with the associated potential to exacerbate any pre-existing tear dysfunctions. A contact lens divides the tear film into pre-and post-lens parts with the thinner pre-lens tear film having a reduced lipid layer thickness [21]. Meibomian gland dysfunction-like changes which can be induced by contact lens wear, may contribute to these tear anomalies [22]. Prime dry eye mechanisms associated with contact lens dryness symptoms in discontinuing contact lens wearers were found to be aqueous deficiency (30%) and evaporative dry eye (39%) [10]. However, 31% of discontinuing patients could not be assigned to one of these dry eye mechanism categories [10], suggesting that their symptoms might be due to multiple tear deficiencies or even to be associated with corneal or lid wiper neuropathy for example. Even so, 23% of symptomatic contact lens patients showed none of the signs normally associated with dry eye problems [10] which raises the possibility that apart from neuropathy, mental health conditions and associated somatisation could increase symptom perception in contact lens wearers as may occur in patients with dry eye disease [23].

### Neuropathic symptoms and signs

A growing body of literature suggests that symptoms of dry eye disease may sometimes be better characterized as neuropathic ocular pain rather than dry eye [24]. For instance, hyposecretion of tears may lead to pathological alterations in corneal nerves [25]. That dry eye disease predisposes to neuropathy is suggested by the findings in a lagophthalmos patient of greater neuropathic changes being found in the lower corneal periphery nerves [25] where evaporative dry eye pathology can be greater due to incomplete blinking-related over-exposure [26, 27]. Kalteniece and co-authors reported greater inferior corneal nerve fibre damage in diabetic patients with diabetic peripheral neuropathy [28], which may also be explained by incomplete blink-related exposure of that area [26]. The significance of incomplete blinking appears likely to be greater according to the degree of contributions from other dysfunctions within the lacrimal functional unit. However, patients with neuropathic pain can experience sensations of dryness even when other tear functions are assessed as normal so that their symptoms appear to be unexplained [29]. Corneal nerve abnormalities found using laser in vivo confocal microscopy in non-contact lens-wearing patients with severe symptoms of pain or photoallodynia, despite the lack of clinical signs on slit lamp examination, suggest a diagnosis of corneal neuropathy [30]. Thus, corneal esthesiometry and in vivo confocal microscopy may be crucially important for appropriate diagnosis of a neuropathic basis for ocular symptoms [31, 32] in contact lens wearers.

Esthesiometry might detect abnormally sensitive corneal nociceptor thresholds and in vivo confocal microscopy might detect corneal sub-basal plexus corneal nerve fibre damage or evidence of repair in the form of abnormal fibre length and the presence of beading and neuromas for instance [33]. Dastijerdi and Dana include nerve sprouts and increased tortuosity as signs of nerve regeneration [25]. Such findings cannot be detected by slit lamp biomicroscopy [30]. In a murine study, exposure to constant wind conditions for 2 weeks after photorefractive keratectomy resulted in a higher number of beads, nerve sprouts and degree of tortuosity being observed in corneal sub-basal nerves [34]. These changes may be indices of high metabolic activity directed to the repair of nerves with the release of neuropeptides by the nerve fibres resulting in the formation of sprouts [34]. In addition, epithelial cells produce soluble factors such as nerve growth factor with associated neurotrophic effects [34]. Any treatment which improves epithelial health may benefit neurotrophic processes. Similarly, activated keratocytes also release nerve growth factor in dry eyes with associated nerve beading and sprouting [34]. The chance of symptoms having a neuropathic basis is complicated by the possibility that any corneal neuropathy observed is associated with another disease such as diabetes [28] or multiple sclerosis [35]. Results from basic and clinical studies demonstrated that neurotrophic factor expression and neurogenesis decrease as a consequence of aging, mental stress and depression [36]. Antidepression treatment can reverse these effects [36]. It appears to be possible that stress and depression, especially in older contact lens wearers could be associated with reduced neurotrophic factor expression and neuropathic changes in the cornea of some patients. However, the central nervous system can also play a role in neuropathic symptom generation without any associated peripheral neuropathy.

### Peripheral and central neuropathic pain

Many medically unexplained disorders are understood to involve an interplay between peripheral and central neuropathological mechanisms [37]. Central sensitization may occur as a result of the continuous activation of nociceptors and the progression of peripheral sensitivity [9, 38]. However, neuropathic pain may have central nervous system origins rather than, or as well as those involving peripheral mechanisms. For example, due to its effect on central pain pathways, Gabapentin (an antiepileptic) has been used successfully to relieve neuropathic pain in patients with severe dry eye [38]. Gamma-aminobutyric acid is the chief inhibitory neurotransmitter in the central nervous system having the principal role of reducing neuronal excitability [39]. Disrupted gamma-aminobutyric acid distribution can result in neuronal hyperexcitability and chronic central neuropathic pain [39]. Persistent ocular pain after topical anaesthetic instillation is a metric of central sensitisation [40]. Neuropathic dry eye symptoms are often associated with numerous comorbid pain conditions [24]. Symptoms of contact lens discomfort might also be associated with comorbid pain conditions that suggest central neuropathy.

### Ocular responses in soft contact lenses which might predispose to neuropathy

The corneal epithelium produces and releases neurotrophic factors to support nerve trophism and healing, and corneal nerves produce trophic neuromediators for the survival, trophism and healing of the corneal epithelium [41]. Could contact lenses disturb epithelial functions sufficiently to predispose the cornea to neurotrophic stress and related neuropathic symptoms? Silicone hydrogel contact lenses have eliminated lens-induced hypoxia for the majority of lens wearers but, especially in the case of extended wear, the turnover of the corneal epithelium is nevertheless slowed by lens wear causing suppression of cell proliferation and migration, as well as decreasing the rate of cell exfoliation [42]. For non-contact lens wearers, physical and chemical agents acting on the ocular surface (such as extreme environmental temperatures, wind, foreign bodies, pollution and noxious chemicals) elicit conscious sensations and reflex motor and autonomic responses (blinking, lacrimation, conjunctival vasodilation) which serve to protect the eye from further injury [43]. However, the peri-limbal conjunctiva and the cornea, may be less likely to be the source of these responses during soft contact lens wear according to the extent that the lens insulates them from ambient stimuli. For example, dry eye symptoms are keyed to the sensitivity of specialised evaporation-sensitive (cold) receptors known as TRPM8 channels which are located in afferent corneal nerve terminals [44]. However, this mechanism is prevented when tear evaporation occurs on the lens rather than the cornea. In addition, the ocular surface is insulated from blink-related lid wiper friction by a contact lens, which may contribute to delayed desquamation of epithelial cells. Disruption of feedback loops between the ocular surface and the lacrimal glands can lead to dry eye disease [7] making it possible that insulation of the corneal surface from normal stimulation may contribute to aqueous deficiency in contact lens wear.

### Lens movement

That soft lens movement can vary over time is indicated by the finding that during the first 25 min after insertion, a significant reduction in soft lens movement was observed but after 8 h, a significant increase in movement could be detected [45]. Increased movement over time may be a function of heightened friction due to lens soiling and reduced lubrication of lid movements over the lens. For example, increased lens movement is associated with increased mucous discharge and lens surface deposition in cases of contact lens-induced papillary conjunctivitis [15]. A study of silicone hydrogel lens wear found no significant difference in lens movement between flatter and steeper base-curve lenses, but the steeper lenses were more comfortable [46]. However, steeper fittings significantly increase the risk of adverse inflammatory events [47] which may be due to or exacerbated by an associated reduction in tear exchange and increased stagnancy of the post-lens tear pool.

### Post-lens tear exchange

The post-lens tear pool in soft lens wear is relatively stagnant and less well understood due to difficulties in evaluation [21]. Limited tear exchange allows metabolic by-products and cellular debris to accumulate under the lens for long periods and increase the risk of inflammation, especially for extended wear fittings [48]. Soft lenses which lose water content by front surface evaporation have the capacity to take fluid from the ocular surface by pervaporation [20] with a similar potential for reducing the volume of post-lens tears and increasing the concentration of metabolic by-products and cellular debris. Under these conditions, the relatively static post-lens tears may alter the epithelial barrier function [48] with the associated possibility of adverse consequences for the corneal epithelium and sub-basal nerve plexus. Production of neurotrophic factors by the epithelial cells may be compromised for instance. Again, soft contact lens wear is known to increase epithelial cell size, thin the central corneal epithelial layer, cause epithelial cyst formation, as well as increase susceptibility to bacterial binding and the risk of infection [42]. Some recovery from any stagnant post-lens tear pool effects could occur during the day if lenses are deliberately displaced to the conjunctiva and then returned to the cornea. However, the potential for recovery is much greater when lenses are removed at the end of the day. The degree of any recovery after removal may depend on the duration of lens-free periods prior to sleep, as well as after sleep and prior to insertion the next morning. These lens-free periods allow normal blink activity and help to restore the health of the ocular surface. Eyes may also benefit if any sleep-related physiological oedema or hyperaemia is allowed to dissipate prior to lens insertion.

### Inflammation associated with soft contact lens wear

Contact lens-induced inflammation can be apparent by slit lamp examination, especially toward the end of the day in association with discomfort [49]. Markouli and co-authors reported finding that both inflammatory mediators and neuropeptide substance P were correlated with several measures of corneal nerve morphology in healthy subjects with mean age of 39 ± 9.9 years [50]. This evidence was interpreted as support for a link between inflammatory mechanisms and nervous systems [50]. Lopez-de la Rosa and co-authors reported that increased levels of substance P found in the tears of symptomatic contact lens wearers suggest that this molecule may be implicated in the development of discomfort [51]. Tissue damage and inflammation of the ocular surface can result in peripheral axonal injuries with the additional release of proinflammatory mediators potentially resulting in the increased sensitivity of peripheral nerves [9]. Examination by in vivo confocal microscopy may detect increased dendritic cell density, size and field (span of cell membrane extensions), which are potential parameters for assessing the inflammatory state of the cornea [52]. The tears of symptomatic contact lens wearers can be found to show increased concentrations of nerve growth factor [53].

Both immune and mechanical processes are associated with increased concentrations of a wide variety of inflammatory mediators in the tears of soft contact lens wearers [54]. Evening peaks of soft contact lens redness were found to coincide with peaks of lens awareness [55]. For contact lens discomfort syndromes, the strongest association with inflammation has been found with lipid degradation products [53]. Lipid degradation and deposition on lens front surfaces may help determine loss of lubricity. Pitenis and co-authors reported in vitro evidence which suggested that frictional hydrogel probe shear stress could be responsible for inducing inflammatory responses in corneal epithelial cells [56]. Inflammatory responses in the lid wiper epithelium may also be driven by increased frictional shear stress when soft contact lens front surfaces become soiled and dry. Mucin balls are spherical, translucent, insoluble, substantially rigid, tear film-derived bodies composed of naturally occurring ocular surface mucins, which form between the back of soft contact lenses and the corneal epithelium [57]. Early-onset mucin ball formation substantially increases the hazard of corneal inflammatory events in subsequent wear with different lens types [57]. For instance, contact lens wear can result in increases in tear concentration of the cytokines IL-6, IL-8, TNF-α and endothelial growth factor [58]. Masoudi and co-authors found that leukotriene B_4_ was the only inflammatory mediator showing an increased concentration in the evening with contact lens wear and suggested the possibility that leukotriene B_4_ concentration could be associated with discomfort during contact lens wear [59]. The extent to which inflammation-related mediators contribute to contact lens discomfort processes may depend on the degree to which they have concentrated in a stagnant post-lens tear pool. For instance, an increased concentration of bacterial exotoxins in the post-lens tears could lead to inflammatory complications if their removal is delayed by tear stagnation beneath the lens [48]. Nomura and co-authors reported an association between corneal neovascularisation and symptoms of dryness among wearers of hydrogel lenses [60].

### Inflammation and neuropathy

In non-contact lens wearers, the nociceptors under normal conditions are protected from the ambient environment by the glycocalyx, mucin layer and tear film [4]. In dry eye disease, the lack of protection for the nociceptors determines the release of neuropeptides (such as substance P) which promote an inflammatory reaction (neurogenic inflammation) [4]. Hyperalgesia in early or mild dry eye disease cases is probably due to this mechanism [61]. In dry eye disease, ocular surface inflammation results in changes in corneal nerve structure and altered responses to mechanical or chemical stimuli [62]. Abnormal morphological changes detected by confocal microscopy (Nidek: ConfoScan 2.0) can be observed in the corneal nerves of patients with aqueous deficient dry eye, with a strong correlation between the morphology changes and degree of aqueous deficiency [63]. Changes in corneal nerve structure might also develop in symptomatic contact lens wear in association with impaired tear functions and inflammation. Increasing conjunctival hyperaemia during contact lens wear and associated warmer pre-conjunctival tears, may result in significantly increased evaporation and greater levels of hyperosmotic symptoms, in addition to having the effect of exacerbating any aqueous deficiency [49, 64]. Such an amplifying cascade of changes is consistent with an increasing risk of symptoms at the end of the day for wearers of contact lenses [49, 64].

### Corneal nerve structure in contact lens wear

Corneal sub-basal nerve fibres are located in the deep subnuclear region of the basal epithelial cell layer, either between the basal epithelial cells and their basal lamina or within cytoplasmic infoldings of basal epithelial cell membranes [65]. Using the Tomey Confoscan confocal microscope (40x/0.75 objective lens) extended wearers, overnight wearers and non-soft contact lens wearers were not found to have corneal nerve morphology or distribution changes from normal controls [66]. In vivo confocal microscopy examinations show that successful long-term (> 10 years) soft contact lens wear and its associated stromal hypoxia and acidosis has no demonstrable effect on keratocyte density [67]. For instance, decreased corneal sensitivity in these contact lens wearers was not found to be accompanied by decreased nerve fibre bundle density [67]. An in vivo confocal microscopy (Confoscan 3.0 (Nidek)) comparison between normal control subjects and healthy subjects fitted with silicone hydrogel contact lenses, did not detect any significant changes in the corneal sub-basal nerve plexus or corneal mechanical sensitivity after six months [68]. The subjects were contact lens naïve and did not have dry eyes, diabetes or other disease that could affect corneal nerves [68]. However, Patel and McGhee examined healthy non-contact lens wearing subjects with laser scanning in vivo confocal microscopy (using a retinal tomographer Heidelberg Engineering Rostock corneal module) and found strong evidence of continuous centripetal movement of identifiable branch points of up to 26 μm per week, with these dramatic pattern changes in the plexus evident over a 6 week period [69]. Also, Lum and co-authors reported an absence of a normal sub-basal nerve whorl-like complex in the eyes of subjects who were regular wearers of orthokeratology design rigid contact lenses [70]. However, any neuropathy in contact lens wearers may be more likely to be found in chronically symptomatic patients and especially those that have been forced to discontinue wear.

### Criteria for identifying corneal neuropathy

Binotti and co-authors reported that corneal nerve sub-plexus alterations can be evaluated according to total, trunk and branch nerve density and length, tortuosity, beading, nerve reflectivity, thickness and microneuromas [52]. Moein and co-authors suggested that microneuromas may serve as a sensitive and specific biomarker for the diagnosis of neuropathic corneal pain [71]. Patients with continuous severe ocular pain for more than a year, with minimal or no ocular surface signs and who were non-responsive to topical lubricants, steroids and cyclosporine were examined using in vivo confocal microscopy by Ross and coauthors [72]. They found that structural and functional nerve anomalies in the form of reduced sub-basal nerve density, aberrant regeneration, microneuromas, reduced or increased sensitivity, associated with keratocyte and inflammatory (dendritic) cell activity, all seem to interact in a cause and effect or a vicious cycle relationship to cause subjective symptoms, especially pain, in the absence of any clinically visible signs on slit lamp examination [72]. Scarpa and co-authors have developed a method of automated classification of confocal microscope images of the corneal sub-basal nerve plexus which achieved 96% accuracy in identifying neuropathy [73]. Could automated algorithms be better able to determine quantitative as well as qualitative neuropathic changes in the corneal sub-basal plexus [74] such as any that could be associated with contact lens discomfort?

### Treatment

Contact lens discomfort may depend on more appropriate choices of lens type fit and care system. Daily disposable lenses may be the most suitable. To the extent that discomfort has a neuropathic basis, treatment for tear deficiencies may support peripheral neurotrophic functions [75]. For example, faster tear loss by evaporation from exposed contact lens surfaces and associated shorter tear break up times suggest that tear supplements and improved blink efficiency [27] may be key approaches.

## Discussion

That factors such as genetic susceptibility variations are believed to play pivotal roles in dictating phenotypic manifestation of ocular pain [72] may help explain why only some cases of lens soiling and unwetting are associated with discomfort symptoms. Bandage contact lenses can be comfortably worn after photorefractive keratectomy when they aid in corneal protection, pain relief and acceleration of the healing process, including re-epithelialization [76]. Chao and co-authors reported that reusable soft contact lens wear was associated with higher concentrations of tear cytokines, more conjunctival staining, and greater conjunctival epithelial metaplasia when compared with daily disposable contact lens wear [12]. However, despite refitting with daily disposable lenses to reduce lens soiling, unwetting and discomfort, discontinuation from wear may still be unavoidable [14]. This finding suggests that eyes could become intolerant to lens wear rather than that contact lenses necessarily acquire features which cause them to become intolerable.

## Conclusions

It is not clear as to whether the potential benefit from physical insulation of the cornea from the ambient environment by the bandage function of a soft contact lens can be undermined by any adverse influences which are associated with stagnant post-lens tears. Chronic wound healing responses in soft contact lens wear, perhaps related to limbal conjunctival trauma, stem cell deficiency and persistent epitheliopathy, as well as one or more immune responses, may contribute directly or indirectly to inflammation and an amplifying evaporative dryness symptom cascade [49]. Symptoms of dryness and discomfort remain a significant problem for too many soft contact lens wearers who subsequently discontinue wear [10, 14]. Contact lens care solutions and accumulated surface deposits on reusable lenses were suggested as explanations for these findings [12]. Daily disposable lenses can address these problems for some patients. That daily disposable lenses were more often associated with discontinuation [14] may be a consequence of that modality being a last resort for patients who are forced to discontinue wearing multiple-use lenses. The advantages of daily disposable lenses, such as avoiding storage solutions and always inserting clean lenses so that end of day soiling is reduced may not be sufficient advantages to allow continuation in such cases. Perhaps prior development of neuropathic changes could reduce the chances of success when switching to daily disposable lenses. Central or peripheral neuropathy could be associated with dryness discomfort symptoms in soft contact lens wearers especially for those that have resulted in discontinuation from lens wear of previously successful wearers.

## Data Availability

Not applicable.
